# A Phase I trial of Non-invasive Ventilation and seizure prophylaxis with levetiracetam In Children with Cerebral Malaria Trial (NOVICE-M Trial)

**DOI:** 10.12688/wellcomeopenres.21403.1

**Published:** 2024-05-20

**Authors:** Kathryn Maitland, Nchafasto Obonyo, Mainga Hamaluba, Emmanuel Ogoda, Christabel Mogaka, Thomas N. Williams, Charles Newton, Symon M. Kariuki, Diana M. Gibb, A. Sarah Walker, Roisin Connon, Elizabeth C. George

**Affiliations:** 1 Department of Infectious Disease and Institute of Global Health and Innovation, Division of Medicine, Imperial College London, London, England, W2 1PG, UK; 2 KEMRI Wellcome Trust Research Programme, Kilifi, Kilifi, PO BOX 230, Kenya; 3 Department of Psychiatry, Warneford Hospital, University of Oxford, Oxford, OX3 7JX, UK; 4 Department of Public Health, Pwani University, Kilifi, Kilifi County, Kenya; 5 Institute of Clinical Trials & Methodology, Medical Research Council Clinical Trials Unit at University College London, London, England, WC1V 6J, UK

**Keywords:** severe malaria, prophylactic anticonvulsants, children, Africa, clinical trial, non invasive ventilation, levetiracetam, biphasic cuirass ventilationn

## Abstract

**Background:**

African children with cerebral malaria and seizures caused *Plasmodium falciparum* are at greater risk of poor outcomes including death and neurological sequelae. The agonal events are severe hypoventilation and respiratory arrest often triggered by seizures. We hypothesised that prophylactic anti-seizure medication (ASM) could avert ‘spikes’ of intracranial pressure during or following seizures and that adequate ventilation could be supported by biphasic Cuirass Ventilation (BCV) which requires no intubation.

**Methods:**

A Phase I trial conducted in Kilifi, Kenya designed to provide data on safety, feasibility and preliminary data on seizure control using prophylactic ASM (levetiracetam) and BCV as non-invasive ventilatory support in children with cerebral malaria. Children aged 3 months to 12-years hospitalised with *P falciparum* malaria (positive rapid diagnostic test or a malaria slide), a Blantyre Coma Score ≤2 and a history of acute seizures in this illness are eligible for the trial. In a phased evaluation we will study i) BCV alone for respiratory support (n=10); ii) prophylactic LVT: 40mg/kg loading dose then 30mg/kg every 12 hours given via nasogastric tube for 72 hours (or until fully conscious) plus BCV support (n=10) and; iii) prophylactic LVT: 60mg/kg loading dose then 45mg/kg every 12 hours given via nasogastric tube for 72 hours (or until fully conscious) plus BCV support (n=10). Primary outcome measure: cumulative time with a clinically detected seizures or number of observed seizures over 36 hours. Secondary outcomes will be assessed by feasibility or ability to implement BCV, and recovery from coma within 36 hours. Safety endpoints include: aspiration during admission; death at 28 days and 180 days; and de-novo neurological impairments at 180 days.

**Conclusions:**

This is a Phase I trial largely designed to test the feasibility, tolerability and safety of using non-invasive ventilatory support and LVT prophylaxis in cerebral malaria.

**Registration:**

ISRCTN76942974 (5.02.2019); PACTR202112749708968 (20.12.2021).

## Plain Language Summary

Unfortunately, children with cerebral malaria continue to have very poor outcomes including severe hypoventilation and respiratory arrest (i.e. breathing is too slow or stops) during hospitalization which is often triggered by seizures. We will explore the potential benefits of a special type of ventilation that applies suction or negative pressure to the chest (meaning keeping children breathing by pushing air in and out of their lungs) in combination with anticonvulsants given before children have had any fits

We will use a device called biphasic Cuirass Ventilation (BCV) that can be used by non-specialists to help children breath. BCV applies both negative and positive pressure to the chest, covering both inspiration (breathing in) and expiration (breathing out) phases of breathing, which is more appropriate for periods of when the breathing is too slow or stops for a period of time.

We will also use an anticonvulsant drug, called levetiracetam to prevent seizures. It has been safely used in Malawian children and shown to improve outcomes. This will be given directly into the stomach via a nasogastric tube (tubes down the nose into the stomach)

The study will be carried out at Kilifi County Hospital, Kenya and plans to enrol 30 children aged 3 months to 12 years with cerebral malaria and a positive malaria test The first ten children with have the BCV device only to assist respiration until they recover from their coma. The next twenty children in the trial will have the BCV device in addition with anticonvulsants given before children have had any fits as a preventive strategy to stop fits.

All children will have regular monitoring during the period of coma/ventilation and will be followed up on days 28 and 180.

The study aims to generate feasibility and safety data to support future trials.

## Abbreviations

AE    Adverse event

ASM    Anti-seizure medication

BCS    Blantyre Coma Scale

BCV    Biphasic Cuirass Ventilation

BUN    Blood urea nitrogen

CM    Cerebral Malaria

CPAP    Continuous Positive Airway Pressure

CRF    Case Record Form

DMC    Data Monitoring Committee

EEG    Electroencephalography

GOR    Gastro-oesophageal reflux

HFNC    High Flow Nasal Cannula

HPLC    High performance liquid chromatography

HRP2 *P. falciparum* Histidine Rich Protein 2

KCH    Kilifi County Hospital

KEMRI    Kenya Medical Research Institute

KWTRP    Kilifi Wellcome Trust Research Programme

ICREC    Imperial College Research Ethics Committee

LVT    Levetiracetam

MAF    Malaria attributable fraction

MRC CTU    Medical Research Council Clinical Trials Unit

NGT    Nasogastric tube

NIV    Non-invasive ventilation

NPV    Negative pressure ventilation

pCO2    Carbon Dioxide (measured by pulse oximetry)

PEEP    Positive End Expiratory Pressure

PK    Pharmacokinetic

PKPD    Pharmacokinetic Pharmacodynamics

RDT    Rapid diagnostic test

SAE    Serious adverse event

SERU    Scientific and Ethics Review Unit

SMAART    Severe Malaria in African children: A Research and Trials consortium

SOP    Standard Operating Procedures

## Introduction

Neurological involvement in African children presenting to hospital with severe *Plasmodium falciparum* malaria is very common. It typically manifests following a short febrile illness with seizures, and altered consciousness, with or without signs of brain-stem involvement. Whilst altered consciousness is common in severe malaria and has reasonable prognosis ^1^, for the most severe form, known as cerebral malaria (defined as an unrousable coma persisting >1 hour post-seizure), outcome is not optimal. Approximately 20% affected children die in hospital ^1^ while others survive with long-term neurological impairments ^2^. The Blantyre Coma Scale (BCS) is the most widely used paediatric classification of impaired consciousness in sub-Saharan Africa. This was developed as a practical tool for children who are too young to speak ^3, 4^. Coma (and hence cerebral malaria (CM)) is classified as a BCS of ≤2 (out of a possible 5) which equates to inability to localise a painful stimulus.

Seizures are the most common neurological complication of acute *P. falciparum* malaria, manifesting as either generalized tonic-clonic or focal seizures ^5^ or clinically silent ‘electrical’ (detected by EEG) status ^6^. Focal seizures may present with subtle clinical symptoms such as excess salivation, eye deviation, lip-smacking, bicycling movements and/or an irregular respiratory pattern ^7^. In a prospective study in Kenyan children with malaria, 38% had a history of seizures or witnessed seizures at admission to hospital. During hospital stay multiple seizures typically occurred in over 50% of these cases, with 22% having prolonged seizures (>30 minutes). Since seizures can occur in febrile non-malarial illnesses in children with coincidental parasitaemia, the malarial attributable fraction (MAF)) was explored in a prospective study ^8^. Children that had two or more seizures in 24 hours prior to hospital admission with malaria had very high MAFs, suggesting that acute malaria was the chief cause of seizures ^8^.

Estimates of the prevalence of neurological sequelae deficits in survivors of cerebral malaria vary ^9– 11^. To address varying definitions, a meta-analysis was conducted including studies which had similar case definitions of cerebral malaria, indicating that sequelae occurred in approximately 11% ^12^. The most common neurological sequelae reported were ataxia (43%), hemiplegia (39%), speech disorders (39%), blindness (30%), cognitive ^2, 13^ and behavioural abnormalities ^14^. Some of the deficits were transient (e.g., ataxia) and fully resolved, whereas others showed improvement over months (e.g., hemiparesis) but did not fully resolve ^15^. A history of previous seizures, deep coma and focal neurological signs observed during admission were independently associated with persisting impairments ^16^.

### Pathophysiology

*P. falciparum* malaria parasite has the unique ability to cause late-stage parasitized red cells to cytoadhere to deep vascular beds, a phenomenon called sequestration. Whilst this can occur during a non-severe infection, autopsy studies have shown that there is intense sequestration of parasitized erythrocytes in other vital organs as well. In cerebral malaria, the pathophysiological process is mediated, partly by intense sequestration of parasitised red cell in the cerebral microvasculature ^17^, rosetting ^18^ and decreased deformability of non-parasitised red cells ^19^. Whether sequestration causes mechanical obstruction and impaired tissue perfusion, or is damaging in other ways (active parasite metabolism, release of toxins, cytokine induction) ^20^ is not known. There is evidence at autopsy of blood-brain barrier disruption, which may contribute to cerebral oedema and brain swelling ^21, 22^. Children dying with cerebral malaria often have clinical signs ^23^ compatible with trans-tentorial herniation and sonographic features of progressive intracranial hypertension during the agonal phases ^24^. Post-mortem studies reveal brain swelling (increased brain weight, compressed ventricles, flattened cerebral gyri) in many children dying with cerebral malaria, but frank herniation at any level of the brain is rare.

### Management of cerebral malaria

Quinine was previously the mainstay of treatment for African children with severe malaria for decades; it remains effective and little drug resistance has been reported on the African continent. It has now been replaced by artesunate as the first line antimalarial for severe malaria following findings of the AQUAMAT trial ^1^. The AQUAMAT trial conducted in 11 centres in 9 countries in Africa compared quinine and artesunate in 5425 children hospitalised with severe malaria. In the intention-to-treat analysis the primary endpoint, in-hospital mortality, was 297/2713 (10.9%) in those receiving quinine treatment compared to 230/2712 (8.5%) in children receiving artesunate - a relative reduction in mortality of 22.5% (95% CI 8.1-36.9); p=0.002 ^1^. The sub-group with the greatest mortality were children with coma (cerebral malaria; 33.6% of all participants). Overall mortality in the group with coma was 359/1825 (19.6%) ^1^ but this was not substantially better in the artesunate-treated children (18%) than quinine-treated children (21%).

### Adjunctive therapies for cerebral malaria

One case series and an uncontrolled trial investigated mannitol and other osmotic diuretics (osmotherapy) as adjuncts for treating brain swelling (cerebral oedema) in cerebral malaria ^23, 25^. Use of mannitol as an adjunct for treating cerebral oedema (osmotherapy) appeared to improve outcomes ^23^. A dramatic improvement was also reported in case series of children receiving urea with dexamethasone ^25^. Nevertheless, a subsequent placebo-controlled trial, conducted in 156 Ugandan children with cerebral malaria, investigating a single dose of 5 ml/kg of 20% (1g/kg) intravenous mannitol infused over 20 minutes demonstrated no important differences in the main outcomes, including time to regain consciousness or death ^26^. Six children had post-mortems of whom five had signs of anoxia and cerebral oedema; the sixth child had signs of acute tubular necrosis at autopsy. Whilst there is good evidence that brain swelling complicates both adult and paediatric cerebral malaria, trials to date have shown no benefit of osmotherapy or use of steroids.

### Seizure management and prophylaxis

The current treatment algorithm for managing children with seizures is a weight-adjusted dose of intravenous or intramuscular diazepam (0.15-0.2mg/kg) to treat a prolonged seizure (> 5 minutes). This can be repeated once and for further seizures intravenous loading of phenobarbitone (15mg/kg) (or phenytoin) is recommended as second-line followed by a maintenance dosage (10mg/kg/day) until the seizures are controlled ^27^. Since seizures are common in severe malaria, particularly in those with cerebral malaria, in whom poor outcomes are common, routine administration of anti-seizure medication (ASM) to prevent the seizures (prophylaxis) has been proposed as an adjunctive treatment for any child who presents with altered conscious (prostration or coma) following severe malaria. The efficacy of this prophylactic treatment awaits further investigation.

### Trials of seizure prophylaxis in severe malaria

A double-blind controlled trial in Thailand, involving both children and adults with cerebral malaria conducted in the early 1990s, randomised participants to receive either a single dose of phenobarbitone or placebo. The trial showed fewer seizures in the phenobarbitone group at 12% (3/24) compared to 54% (13/24) in the placebo group (p = 0.006) ^28^. Owing to the small sample size there was no statistical evidence of an effect on mortality. However, deaths were higher the phenobarbitone arm, occurring in 8 (33%), all of whom were in deep coma at admission versus 5 (21%) in the placebo, none of whom were in a deep coma on admission (p=0.5). Based on this small study, the authors recommended that guidelines should advocate that all patients with cerebral malaria should receive a single intramuscular loading dose of phenobarbitone.

### Trials of seizure prophylaxis in African children with cerebral malaria

***Phenobarbitone***

A large single centre paediatric placebo-controlled trial (N=340) conducted in Kenyan children with cerebral malaria was designed to provide the evidence for this new recommendation following the Thai study. Children with cerebral malaria were randomized to a single intramuscular dose of phenobarbitone (20mg/kg) versus placebo. As expected the phenobarbitone group had fewer multiple seizures (defined as three or more seizures of any duration) than the placebo group (18 [11%] vs 46 [27%], odds ratio 0·32 [95% CI 0·18–0·58]), but mortality was more than double in the phenobarbitone group (30 [18%] compared to 14 [8%] placebo deaths; relative risk 2·39 [95% CI 1·28–4·64]) ^29^. The main mode of death in those receiving phenobarbitone was respiratory arrest. Following this trial the guidelines changed and no longer recommended phenobarbitone prophylaxis in cerebral malaria.

***Fosphenytoin***

A further placebo-controlled trial of seizure prophylaxis conducted in Kilifi, Kenya, involving children with non-traumatic encephalopathy (with a large subgroup of cerebral malaria, n=110) investigated the use of fosphenytoin. Fosphenytoin was considered as a superior for seizure prophylaxis, because it has been used in traumatic brain injuries with minimal cardiorespiratory side effects. This trial was terminated due to slow recruitment/futility. At least one seizure (monitored clinically and by electroencephalogram) occurred in 33/83 (40%) children receiving fosphenytoin versus 32/88 (36%) receiving placebo (P=0.73) ^30^. In the cerebral malaria sub-group, 20 (37%) children in the fosphenytoin group had seizures; which was similar to the that in the placebo group (21, 38%). Overall, 18 children treated with fosphenytoin (21%) died compared with 15 in the placebo group (17%) (P=0.49). In survivors, occurrence of neurological impairments at 3 months was similar (10%) in both groups ^30^.

***Levetiracetam (Keppra)***

Owing to its favourable properties, levetiracetam (LVT) is increasingly the drug for choice in children with status epilepticus ^31^. However, the cost of intravenous formulations have been prohibitive (including at the time the NOVICE trial was designed) and thus, some investigators have considered the oral formulation (dosed via a nasogastric tube). LVT has favourable enteral bioavailability, and thus the oral formulation could be adopted into future guidelines if shown to result in improved neurological status in those surviving cerebral malaria.

An open-label randomised trial of enteral levetiracetam (LVT) was designed to evaluate pharmacokinetics, safety and efficacy of orally dosed LVT compared to parenteral phenobarbitone in Blantyre, Malawi in children with cerebral malaria ^32^. The initial dose-escalation study planned to investigate 4 strata of LVT doses starting with a standard dose (40 mg/kg load, then 30 mg/kg every 12 hours),titrated upward until 75% of subjects had attained seizure freedom. Of the 32 patients anticipated only 7 were enrolled, all received LVT 40 mg/kg load plus 30 mg/kg every 12 hours and all achieved seizure freedom. Consequently, enrolment was stopped as dose escalation was not justified.

Children were eligible for the planned randomised controlled trial if they were aged 24–83 months, with BCS ≤2, were *P. falciparum* positive (slide or RDT) and had a history of a seizure in the proceeding 24 hours. Children with a plasma creatinine >2mg/dl were excluded as were children on HIV or TB medications. The randomised trial planned to enrol 60 children and randomise these eligible children (on a 1: 1 ratio) to orally dosed LVT (at 40 mg/kg load, then 30 mg/kg every 12 hours) versus phenobarbital (at 20 mg/kg load, then 5 mg/kg every 12 hours up to 72 h). The primary endpoint was minutes with seizure activity (recorded by EEG) within 72 h post-treatment allocation. Secondary outcomes included seizure freedom, treatment failure requiring cross over, coma duration, neurologic sequelae. Children in the LVT arm in the trial had additional sampling for the PKPD analysis. Following an interim analysis, which recorded more adverse events (largely respiratory depression in the phenobarbitone arm), children randomised to this arm subsequently only received phenobarbitone as treatment for seizures (clinical or electrographic) that remained unresponsive to diazepam or paraldehyde.

*Pharmacokinetics and Pharmacodynamic studies:* Thirty children provided data for the pharmacological studies which demonstrated that orally-dosed LVT was rapidly absorbed and well-tolerated. Within 4 h of the initial dose, 90% of children reached therapeutic levels (defined as > 20 μg/mL). LVT clearance was found to be lower in patients with higher admission serum creatinine ^32^.

*Phase II RCT* In the randomised trial 23 children were randomised to LVT, 21 to phenobarbitone (13 to prophylaxis and 8 (post trial-amendment) to adhere to treatment guidelines only (2 received phenobarbitone). No differences were found for the primary endpoint (seizure freedom, minutes of seizure events, coma duration, neurologic sequelae or death). Adverse events attributed to phenobarbital included respiratory suppression, aspiration and prolonged somnolence occurred in 4 children, but none of these were recorded in the LVT arms. Phenobarbital was discontinued in 3/15 due to respiratory side effects. The authors concluded there is sufficient evidence provided by the trial to recommend LVT as a safe option for seizure control ^32^.

### Interventional strategies considered by the SMAART consortium

The consortium “The Severe Malaria in African children: A Research and Trials consortium (SMAART)” recognized that prophylactic ASM could avert ‘spikes’ of intracranial pressure during repeated seizures; yet these come at a risk of hypoventilation and respiratory arrest following use of some first generation ASM ^29^. In high-income countries, management of cerebral malaria incorporates mechanical ventilation; but this is not available in the majority of resource-limited hospitals in Africa. A Phase I study would be the first step in addressing whether existing technologies for non-invasive ventilation plus prophylaxis for seizures could be feasible and safely used in children with severe malaria. Furthermore, whilst the potential for respiratory depression with LVT appears less of a concern than with phenobarbitone, there is still a need to consider this alongside non-invasive ventilation (NIV) as joint strategy to improve neurological status in surviving children (in a future Phase III trial).

### Non-invasive ventilation: Biphasic Cuirass Ventilation

Unlike Continuous Positive Airway Pressure (CPAP) or high flow nasal cannula oxygenation (HFNC), which apply positive pressure throughout the entire respiratory cycle in patients breathing spontaneously, Biphasic Cuirass Ventilation (BCV) applies both negative and positive pressure to the chest, covering both inspiration and expiration phases of breathing, which is more appropriate for periods of hypoventilation and apnoea. BCV works using a clear plastic shell called a Cuirass. The Cuirass is lightweight and has a foam seal that maintains an airtight fit around the patient. It is very comfortable to wear. It is available in 11 different sizes, ranging from babies to adults. The Cuirass is placed on the patient and connected to a United Hayek power unit. BCV can be used both in the hospital setting or at home. It does not require intubation and is thus clean and hygienic (BCV has been reported to be very comfortable to patients. The seal of the shell is made of very soft foam, the Cuirass is very, very light and it is designed to fit like a glove. As the Hayek power units automatically compensate for leaks, the Cuirass does not need to be applied tightly. The Device has been certified for use since 2000 and holds an EC Quality Assurance System Certificate (Number GB00/52285) meeting the requirement for the directive 93/42/EEC. From the website: https://hayekmedical.com/what-is-bcv/.

Several studies have shown that effective ventilation can be achieved with BCV in adults without underlying lung disease and in those requiring ventilatory support for acute respiratory failure ^33, 34^. In a retrospective case series of 233 children treated with NPV using BCV at a single paediatric intensive care unit for acute respiratory failure of varying aetiologies (bronchiolitis in 70%), the majority (70%) responded to NPV therapy, defined as not requiring escalation to any form of positive-pressure ventilation ^35^. Effective use of BCV in the paediatric setting has also been described in the management of hypoventilation due to neuromuscular disease ^36^. In anaesthesia, BCV has been used for peri-operative ventilation during complex airway surgery ^37, 38^. Whilst there is a small potential risk of aspiration, a recent observational study in 233 patients treated with BCV for acute respiratory failure reported gastro-oesophageal reflux (GOR) in only 2 patients (0.9%). GOR was defined as 'any providers’ documentation of a witnessed aspiration event, emesis, or feeding intolerance that occurred in subjects receiving enteral feeding while on NPV support. Even with this broad definition of GOR, the approximate baseline risk of aspiration with BCV was <1% ^35^.

### Pharmacology of Levetiracetam for seizure prophylaxis

LVT has a rather unique preclinical profile since its pharmacokinetic profile closely approximates the ideal characteristics required of an ASM ^39^. These include good bioavailability when given orally, and achieving rapid steady-state concentrations with linear and time-invariant kinetics. In addition, it has minimal protein binding and requires minimal metabolism, thus is not dependent on the hepatic cytochrome P450 system. Furthermore, unlike other ASMs, LVT does not inhibit or induce hepatic enzymes. Thus, the risk of clinically-relevant drug-drug interactions (e.g. reducing bioavailability of other ASMs) with LVT is very low owing to its lack of hepatic metabolism, and its lack of protein-binding. Moreover, distinct from other available ASMs, LVT has a wide margin of safety when used clinically. The main clinical side effects reported are drowsiness and postural dizziness (in ambulant populations) and a very small risk of hypotension at higher doses. However, a study of its efficacy and safety in critically ill children with status epilepticus or acute repetitive seizures found no evidence of adverse events including cardiovascular instability ^39^.

LVT is currently under investigation in the UK as a preferred second-line ASM for children presenting to emergency rooms with status epilepticus to the current standard treatment, phenobarbitone or phenytoin ^40^. The absolute oral bioavailability of LVT is nearly 100%. The extent of absorption is independent of dose and is not affected by food intake. Peak plasma concentrations (Cmax) are achieved at around one hour and then decline to baseline within 48 hr following a single oral ingestion. Following a 1000 mg dose of LVT in adults (repeated 12 hourly), Cmax is typically 31 and 43 mg/mL respectively. Steady-state concentrations are generally attained after 2 days of repeated twice-daily dosing. In multiple dose-ranging studies, LVT has exhibited predictable, linear, and dose-proportional steady-state pharmacokinetics ^39^. Owing to its favourable properties, levetiracetam is increasingly the drug of choice in children with status epilepticus ^31^. However, the cost of intravenous formulations at the time the NOVICE trial was designed were prohibitive. As described above it has been shown to potentially have more favourable outcomes in children with severe malaria; although more work is required to optimise the dose in different settings.

## Protocol

### Trial registration

This trial has been registered on the ISRCTN registry protocol number ISRCTN76942974 (registered on the 5 ^th^ February 2019) and on the Pan African Clinical Trials registry protocol number PACTR202112749708968 (registered on 20th December 2021).

### Study hypothesis

We hypothesize that negative pressure ventilation (NPV) for preventing respiratory arrest in combination with prophylactic ASM may prevent or reduce intracranial hypertension by targeting the cerebral vasodilation, and is thus a promising combined strategy to avert deaths and neurological impairments from cerebral malaria.

### General objectives

We aim to conduct a Phase I trial to demonstrate the feasibility and safety of non-invasive ventilatory support in cerebral malaria (Blantyre coma scale ≤2) and history of seizures to generate preliminary data on seizure control and neurological status in those surviving to 28 and 180 days.

***Specific objectives***

**(i)**  To initially examine whether it is safe, feasible and practical to implement a protocol for the use of NIV with BCV, with or without the use of Hi-Flow AirVO2 technology (to treat hypercarbia (PCO _2_))
**(ii)**  To implement our refined-protocol for BCV use in cerebral malaria in addition to prophylactic ASM (LVT) to reduce the complications of cerebral malaria.
**(iii)**  To estimate the occurrence of adverse events using BCV, including episodes of bradypnoea or apnoea; hypoxaemia (oxygen saturations <92%); number of episodes of hypercarbia (pCo2>45mmHg since this will worse brain swelling), and number of aspiration events (given that all children will routinely having nasogastric tubes and regular suction)

**Site:** High dependency ward in Kilifi County Hospital, Kenya.

**Study design**: Phase I trial to generate feasibility, safety and preliminary data on seizure control and survival to 28 days of non-invasive ventilatory support and seizure prophylaxis in cerebral malaria.

**Study Population:** 30 children hospitalised with cerebral malaria

***Inclusion criteria***

1. Aged 3 months and 12 years (up to 40 kg in weight for paediatric dosing of LVT) admitted to the paediatric ward.
2. Current or recent evidence of *P. falciparum* malaria (slide or rapid diagnostic test (RDT) positive)
3. Blantyre Coma Score2 or less that persists even after correction for concurrent hypoglycaemia (defined as glucose <3 mmol)
4. History of seizures in this illness
5. Guardian or parent willing and able to provide consent

***Exclusion criteria***

1. Known cerebral palsy or significant neuro-development delay (which will affect endpoint assessment)
2. Skin disease or burns preventing use of the BCV
3. Respiratory or cardio-respiratory arrest prior to enrolment
4. A comorbidity which clinician believes has a significant risk of poor outcome e.g. malignancy, end-stage renal failure, major cardiac condition

### Sample size determination

This is a Phase I trial designed to test the feasibility, tolerability and safety of using non-invasive ventilatory support in cerebral malaria. We plan to initially enrol 10 children with CM and a history of seizures (see inclusion criteria above) as this selects a high-risk group with ~90% risk of further seizures who will all require first line (diazepam) and many will require second-line (phenobarbitone) anticonvulsants, all with an inherent risk of respiratory depression or aspiration (15% in the control group of the Malawi trial Trials.gov (Trial reference NCT01982812)).

Following this initial phase the next 10 children will receive a neuroprotective strategy of BCV plus LVT 40 mg/kg loading dose and 30mg/kg every 12 hours given via nasogastric tube for 3 days or until coma resolution.

If this reduces seizures that are short lived and are not clinically-relevant (so 80% do not require additional ASM (based on the judgement of an independent Data Monitoring Committee, i.e. that do not require addition ASMs with minimal adverse events), then an additional 10 children will be studied using LVT at this dosage, without dose escalation.

If this fails to adequately control seizures, the neuroprotective strategy of BCV plus a higher dose (150%) of LVT will be studied, namely 60 mg/kg loading dose and 45mg/kg every 12 hours given via nasogastric tube for 3 days or until coma resolution.

## Study procedures

All children admitted with suspected malaria and impaired consciousness (BCS <2) will be screened for study inclusion by the paediatric triage/admission team. Prospective written, informed consent will be sought from parents or guardians of children who are considered to be sufficiently stable. Parents or guardians will be given an information sheet in their usual language containing details of the BCV/LVT study. These will be translated into to local languages, and then back translated (to ensure details are correct) prior to piloting before the initiation of the trial. The sheet will be read aloud to those who are unable to read. Parents and guardians will be encouraged to ask questions about the trial prior to signing the consent form. Consent will include permission for the collection of clinical data and for aetiological investigations. The rights of the participant to refuse to participate without giving reasons must be respected. A number of children will present as emergencies where delay in study enrolment, and thus treatment, will not be practical or indeed humane. We will use a modified form of deferred consent that we developed, received ethical approval for, and used in the FEAST trial. It proposes to use a ‘two-stage’ consent process in this circumstance ^41^. Verbal assent will be sought from parents or guardians by the admitting medical team, if it is considered that the full consent process would significantly delay enrolment, and consequently could be detrimental to the child’s health. Full consent will be sought once the child’s clinical condition has been stabilized. Caregivers will be provided with a brief verbal description of the trial and will be given the opportunity to “opt out” of clinical research. The clinician will later sign the verbal assent form, which will be filed with the consent form. If consent is withdrawn later no data from the subject will be used. A social science study of the consent processes used in FEAST found this to be acceptable to parents and health-care workers ^42^. As in the FEAST trial, if following an assent process a child died prior to full written consent, full consent would not be sought. This was process of emergency consent was approved (by multiple ethics research committees) for FEAST and has been subsequently approved for use in a transfusion trial in Uganda and Blantyre (TRACT) ^43^.

### Trial treatments

We will study 30 children. This will be done sequentially (10 children with usual care + BCV; then 10 children with BCV given 40 mg/kg loading dose of LVT and 30mg/kg every 12 hours; and the final 10 children (with or without BCV) will receive LVT but the dose will depend on whether adequate seizure control (see above) has been achieved in the previous 10 children as follows. Firstly, if seizure control is adequate then an additional 10 children will be studied with BCV plus LVT at the same dosage or secondly, if seizure control is not adequate then LVT at a higher dose will be given 60 mg/kg loading dose and 45mg/kg every 12 hours.

The first 10 children in the study will have the usual care for cerebral malaria with the addition of BCV. This will allow us to assess feasibility and tolerability and to provide initial refinements for its use. The BCV ventilation intervention period **will be from study enrolment** until when a child can respond to stimuli (Blantyre coma scale 3 or more) sustained over a period of 4 hours.

Feasibility will be judged by the ability of BCV to safely institute negative pressure ventilation, and to support respiration during periods of hypoventilation or apnoea. In addition, we will assess whether children are able to tolerate the use of BCV (judged by children remaining comfortable during transition from coma to a semiconscious state) and whether we are able to clear any secretions by regular suctioning (if this is judged to be a problem). Additional practical considerations will be considered such as whether BCV interferes with monitoring the child and time required by the nursing staff to implement and maintain its use.

This will then be followed by a second phase where we will implement a neuroprotective strategy or treatment bundle: where prophylactic ASM will be used in combination immediately following feasibility studies of use of BCV to provide negative pressure ventilation (NPV) +/- AIRVO2 High Flow (children with BCS≤2). We will use LVT at an initial loading dose of 40 mg/kg followed by 30mg/kg every 12 hours. This will be given via nasogastric tube for 3 days or until coma resolution.

### Sampling strategy

Eligible children will have been admitted to High Dependency Unit in Kilifi County Hospital where children can be closely monitored. Here, once malaria and coma status is verified the parents can be approached for consent and, if they agree to participate, enrolled into the trial. Admission blood will be tested for haemoglobin, lactate, blood urea nitrogen (BUN), glucose, and when possible blood gases (including pC02 and base excess) ^44^. In addition, we will test for malaria status, and collect usual admission blood samples (full blood count, clinical chemistry, lactate), blood cultures and store plasma on day 0. Management and outcome data will be collected (clinical parameters and recovery, developmental assessment, number of transfusions, and use of drugs (specifically ASMs, paracetamol and antibiotics, date of discharge or in-hospital death)). Contact and locator data will be recorded so that children can be invited by phone of face-face for followed at day 28, day 90 and day 180. This system has worked very successfully in our transfusion trial (TRACT) ^43^ with 98% and 95% 90-day and 180-day retention respectively. No additional blood storage will occur after Day 0.

## Clinical assessment

Vital signs will be obtained from bedside patient monitor (temperature, heart rate, respiratory rate, blood pressure, oxygen saturation; conscious level, PCO _2_ monitoring). During admission, clinical seizures will be timed and recorded. Further seizures will be treated in a standard way as per local guidelines/protocol. Efficacy will be evaluated by the reduction in the number of clinical seizures; the number of additional ASM required and neurological status following survival. Safety (see endpoints section below) will be monitored using the number of aspiration events; the number of episodes of hypercarbia requiring AIRVO2 use; the number of episodes of hypotension (a potential side effect of LVT).

Children will be reviewed daily until discharge and followed up at day 28, day 90 and day 180 for neurological or neurodevelopmental impairments (see Table 1). Non-compliance is limited by the intervention being administered by clinical teams during admission with careful stopping rules if a child is not able to tolerate the device or develops a complication including aspiration (secondary safety endpoint). Children lost to follow-up before day 180 will be traced for vital status (permission requested within consent) using locator data and multiple contact phone numbers recorded before discharge.

**Table 1.** Patient assessment.

Assessment Time											
Procedure	Adm	1hr	2h	4hr	8hr	12h	24hr	Bi daily until discharge	Day 28	Day 90	Day 180
Clinical assessment	X	X	X	X	X	X	X	X	X	X	X
Laboratory tests *	X				X		X				
Stored blood **	X										
Neurological exam	X									X	X

* Full blood count, clinical chemistry, lactate, glucose, venous blood gases (including pC02 and base excess), blood culture (admission only) **For quantitative plasma HRP2 assessment

## Trial outcome measures

### Primary endpoint

Cumulative time with clinically detected epileptogenic seizure activity over 36 hours.

### Secondary endpoints

Feasibility will be assessed by ability to implement/operationalise the BCV for use on the high dependency ward in Kilifi County Hospital (assessed by whether this can generate negative pressure ventilation as per specification by the Hayek recommendations and averts respiratory safety endpoints).

### Safety endpoints

Aspiration (sudden decrease in oxygen saturations, and/or denovo presence of coarse chest crepitations, with evidence of gastric reflux/aspirate in the oropharynx).

Episodes of hypercarbia (defined as pCO2 level of greater than 45 mmHg).

Episodes of bradypnoea and hypoxaemia (oxygen saturation <92%) in a child with unrousable coma (Blantyre coma scale≤2) (bradypnoea defined as <10, 15 or 20 breaths/minute over 3 minutes for those aged <6m, 6–36m and >36m respectively).

Development of hypotension (defined as systolic blood pressure <50 mm Hg in children younger than 12 months; <60 mm Hg in children 1–5 years and <70 mm Hg in children olderthan 5 years of age). Use of additional ASMs. Neurological sequelae at day 180, Day 28 and day 180 mortality. Re-admission to hospital through day 180. Serious adverse events through day 180. Grade 3/4 adverse events through day 180. Length of initial hospitalisation. De-novo evidence of neurological impairments will be ascertained using a modified Kilifi Developmental Index ^45^ (which we have adapted to use for the COAST trial) ^46^.

Serious adverse events (SAEs) will use the standardized definitions. SAEs will be independently reviewed in real-time by the DMC. Adverse events will be graded following the Common Toxicity Criteria v5.0:

( https://ctep.cancer.gov/protocoldevelopment/electronic_applications/docs/CTCAE_v5_Quick_Refe rence_5x7.pdf).

### Interim reviews

The Data Monitoring Committee (DMC) will review data from the first 10 children enrolled to receive LVT. If the DMC consider that it reduces seizures that are short lived and not clinically-relevant i.e. that 80% do not require addition ASMs then an additional 10 children will be studied using LVT at this dosage. If it fails to adequately control seizures, the neuroprotective strategy of BCV plus LVT for those in coma a higher dose (150%) will be studied, namely a 60 mg/kg loading dose followed by 45mg/kg every 12 hours given via nasogastric tube for 3 days or until coma resolution. In addition to assessing whether or not the 150% higher LVT dose should be tested, the DMC will also review the accumulating safety data after each adverse event potentially related to the intervention (as defined below) occurs, using a Bayesian continuous monitoring approach for safety. The Bayesian approach uses information from other studies to define a prior distribution for an expected rate of an adverse event before the trial starts, thus formally inferring available knowledge on safety on children with cerebral malaria in these settings. After each child completes 72 hours from enrolment, they will be defined as having had a clinically important adverse event potentially related to the intervention or not. This information is used to iteratively update the prior distribution and to estimate a posterior distribution. The posterior distribution is then used to estimate the probability of the clinically important adverse event rate being higher than a defined threshold and is continually updated throughout the trial. If the probability reaches a specific pre-defined value, then the DMC is prompted to consider stopping the trial. Thus, the decision rule to be used by the DMC to consider stopping the trial is based on a high posterior probability (τ) that the estimated percentage of children with clinically important adverse events is above a threshold R (equivalently: Pr(P _events_>R | data)>τ). Thus, this way of monitoring uses both available knowledge from previous studies and accruing data from the ongoing trial to inform decisions.

The clinically important adverse events potentially related to the intervention for a Bayesian monitoring approach to be considered by the DMC in this Phase I trial will be an aspiration or respiration depression event (defined as a sudden decrease in oxygen saturations, and/or de-novo coarse chest crepitations, with evidence of gastric reflux/aspirate in the oropharynx). As the Phase II trial comparing levetiracetam to standard of care (control arm) in Malawi had not been reported when this protocol was designed we used the data posted on Trials.gov for the to inform the anticipated event rates. This reported that there were 3/21 events (15%) in the control arm (Trials.gov: NCT01982812). Thus, this has informed a beta prior distribution of (0.23, 1.32) with mean 0.15 (i.e. 15% children experiencing an event) and variance 0.05. We have defined a stopping threshold of 20%, as a previous case series identified very few aspiration events (<1%) in children with acute respiratory failure using the BCV machine ^35^. The posterior probability τ was empirically set at 0.95 for the evaluation of this rule ^47^. Thus, according to the derived Bayesian decision rule, enrolment will be stopped if the posterior probability of the number of clinically important adverse events being above the target threshold (20%) is higher than 0.95.

### Clinical management

Members of the clinical team and study team will all receive pre- and peri-trial training on the management of severe malaria and the use of BCV. A manual of operations for the trial will be available for the study team, to anticipate and troubleshoot any potential issues. This is the approach that was taken for training of AirVo2 in the COAST trial and this ensured adherence to the protocol ^46^. Our programme has a dedicated high dependency ward with a long track record of research and care for children with cerebral malaria and has the relevant expertise in the management of this condition. The usual protocol for management of seizures will be followed.

One potential risk of BCV use is aspiration; however this risk is minimal (<1%) ^35^, and further all children with coma routinely have nasogastric tubes placed (to drain gastric contents and secretions) and regular suction (oropharynx) which would apply to all children enrolled in this study as this is part of usual practice. The other potential risk is retention of carbon dioxide, which will be explored in detail in this Phase I study, using non-invasive end-tidal capnography (E’CO2) monitoring. High measures of E’CO2 will be verified by venous blood gas measurement (PCO2). If indicated (PCO _2_ >45 mmHg) then AIRVO2 will be started to wash out CO _2_ since hypercarbia can worse brain swelling. Continuous capnography is increasingly being used across Africa and supported by the Safe Surgery initiative ^48^, as it provides information on the efficiency of respiratory cycle (in non-intubated as well as intubated patients) which is an important parameter to capture in children with cerebral malaria as well as providing a continuous reading of expired CO2. As transcutaneous monitoring is not possible (or affordable in the long-term) our group (and external expert collaborators) prefer use of capnography by regular sampling expired gas from nasal cannulae. All abnormal measurements will be checked against a venous blood gases (0.2mls blood) as an alternative method of measurement. Finally, bag and mask ventilation is also available for children developing short term apnoea who are not ‘rescued’ by BCV.

Children will initially receive parenteral antimalarial treatment (artesunate), followed by an oral course of artemisinin combination therapy, on day 3 (or when the child can safely take and retain oral feeds and fluids). All trial patients will receive intravenous antibiotics. Intravenous maintenance fluids will be given at a rate of 4ml/kg/hour until the child is able to drink and retain oral fluids. Antipyretics and treatment for hypoglycaemia and other treatments will be given as clinically required and will be administered according to nationally agreed protocols. Children with Hb <4g/dl (or Hb <6 g/dl and respiratory distress) will be transfused with 20mls/kg of whole blood.

### Trial withdrawal

In consenting to the trial, patients are consenting to trial treatment (BCV with or without prophylactic anti-convulsants), data collection and follow-up. If a carer wishes to withdraw their child from trial treatment, the investigator will explain the importance and benefits of follow-up, and the value of allowing routine clinical data to be used for trial purposes. Patients/parents are free to discontinue any part of their trial treatment or discontinue from follow up. After the participant has entered the trial the clinician remains free to give alternative treatment to that specified in the protocol at any stage if he/she feels it is in the participant’s best interest, but the reasons for doing so should be recorded. In these cases, the participants remain within the study for the purposes of follow-up and data analysis. All carers and participants are free to withdraw at any time from the protocol treatment without giving reasons and without prejudicing further treatment. If they do not wish to remain on trial follow-up, however, their decision must be respected and the patient will be withdrawn from the trial completely.

### Protocol treatment discontinuation

An individual patient may stop treatment early or be stopped early for any of the following reasons:

▪   Unacceptable toxicity or adverse event

▪   Intercurrent illness that prevents further treatment

▪   Any change in the patient’s condition that justifies the discontinuation of treatment in the clinician’s opinion

▪   Withdrawal of consent for treatment by the patient or parent.

Participation in the trial is entirely voluntary, and parents, carers or older children may choose to discontinue the trial treatment at any time without penalty or loss of benefits to which they are otherwise entitled. Although not required to give a reason for discontinuing their trial treatment, a reasonable effort should be made to establish this reason while fully respecting the patient's rights.

Patients should remain in the trial for the purpose of follow-up wherever possible (unless the patient withdraws their consent for follow-up). If a patient withdraws from the trial, the medical data collected during their previous consented participation in the trial will be kept and used in analysis. This will also apply to parents/carers who withdraw from the trial after assent, that have not completed the deferred consent process. Consent for future use of stored samples already collected can be refused when leaving the trial early (but this should be discouraged and should follow a discussion). If consent for future use of stored samples already collected is refused, then all such samples will be destroyed following the policies of the institution.

### Trial monitoring

The trial will be monitored by the Clinical Trial Facility in Kilifi which oversees standards and quality of all trials conducted through the KWTP and through its monitoring systems and standard operating procedures are organised to ensure that all sites can be monitored with equal independence and rigor. All monitors will be appropriately qualified and trained. At each monitoring visit the monitors will:

▪   verify completeness of Trial Master File

▪   confirm adherence to protocol

▪   review eligibility verification and consent procedures

▪   look for missed clinical event reporting

▪   verify completeness, consistency and accuracy of data being entered on case report forms (CRF)s

▪   evaluate drug accountability

▪   provide additional training as needed

The monitors will require access to all patient medical records including, but not limited to, laboratory test results and prescriptions. The investigator (or delegated deputy) should work with the monitor to ensure that any problems detected are resolved.

### Data management

All clinical and laboratory data will be recorded in the CRF and stored with a unique serial number identifier. Data will be entered onto OpenClinica. All data will be regularly backed up and backup copies stored both on and off site. Paper records will be archived in locked cabinets. These cabinets will have limited access with prior authorisation. All data will be partially- anonymized prior to presentation or publication of any results. Archive documents will be sent for long term storage (10 years) at an appropriate facility according to national policies.

### Confidentiality

Participant’s identification data will be required for the registration process. All clinical and laboratory data will be recorded in the CRF and stored with a unique serial number identifier. Information will only be made available to those caring for the child and those directly involved with the study. Data will be entered onto OpenClinica (FDA approved, web-based application). All data will be regularly backed up and backup copies stored both on and off site. Paper records will be archived in locked cabinets. These cabinets will have limited access with prior authorisation. All data will be partially- anonymized prior to presentation or publication of any results. All clinical data will be held confidentially, and personal identifiers will be removed before analysis of the data and presentation of the results.

### Data sharing

After completion of the study, requests for data access from researchers outside the study team will be considered by a subgroup of the Centre Scientific Committee (Data Governance Committee), and where indicated, requestors will be asked to develop scientific protocols for approval of secondary analyses. The potential to share data will be included in the participant Information and Consent Form.

### Statistical analysis

Clinical data will be summarised using means and medians were appropriate for continuous data depending on the distribution. Analyses will follow intention-to-treat. Primary and secondary endpoints will be described using means or medians or proportions. For the primary endpoint (cumulative time with epileptogenic seizure activity over 36 hours), children who die without recovering from coma will be imputed to have a cumulative time of 36 hours (maximum expected coma duration). As this is a Phase I trial no subgroup analyses are planned.

## Ethics statement

Ethical approval has been obtained from Kenya Scientific and Ethics Review Unit (SERU), Nairobi Kenya on 13 ^th^ July 2018 (protocol number RES/7/3/1), Imperial College Research Ethics Committee (ICREC) on 19 ^th^ June 2018 (protocol number 18IC4511). The trial was registered on the ISRCTN registry protocol number ISRCTN76942974 on the 5 ^th^ February 2019 and on the Pan African Clinical Trials PACTR number PACTR202112749708968 20th December 2021.

## Safety

The study will be performed in patients who may potentially benefit from the treatment.

The trial will be recruiting patients with severe illness and likely a high mortality rate. At the start of the trial, the site will receive appropriate training on the use of the machine and will have 2 dedicated clinicians. The monitors and the BCV has an established safety and acceptability record in a wide range of patient populations, including young children.

## Risks

One potential risk of cerebral malaria is aspiration, where saliva or vomit goes into a child’s throat and makes them start to choke. We will monitor very closely for this as all children with coma routinely have nasogastric tubes (tubes down their noses) and regular suction and this would continue in this study. We do not think that BCV device will increase this risk as it was very small in other studies where it has been used (<1%). The other potential risk is children getting too much carbon dioxide in their blood. This will be monitored very carefully in this study. If indicated, we will optimize the BCV protocol by including the co-use of non-invasive high flow air/oxygen by AIRVO2 via nasal cannulae. Both methods of ventilatory support are widely available, and therefore their inclusion would not affect generalisability.

## Benefits

All patients will be closely monitored so that clinical deteriorations can be identified at the earliest opportunity and appropriate therapy initiated. In general, the high dependency ward at Kilifi County Hospital has considerable experience with this population and this will serve to minimise the risks to the patients and the trial. Prior to the start, the dedicated study teams will undergo detailed training on general management of severe malaria and its complications and receive very detailed training management. Hayek have a demonstration suite in London where one or two of the trial team (KM and NO) will visit and be fully trained before the start of the clinical trial. We will have a live version of the manual of operations which will provide clear and continuously updated guidelines on how to implement the BCV and what signs of deterioration to look for and how to treat these. We believe this will afford all children enrolled in the trial with a higher quality of care. All routine non-trial medications required by the hospital to treat the child will be made available.

The parents or guardians for the children will be asked to return for a follow up clinic visit at day 28, day 90 and day 180, and thus will be offered continuing care for concurrent illness, including any investigations or blood tests that are clinically indicated.

## Plans for dissemination of the study outcomes

This is a Phase I trial of an emergency intervention where our engagement has been at a scientific rather than public/community level. When the results are available, a summary will provide key trial findings and the next steps. If a larger platform trial arose from this study, we aim to develop a dedicated and informed engagement strategy as part of this future trial.

## Discussion

For children hospitalised with severe malaria, despite implementation of fast-acting effective antimalarial drugs (artemisinin-based combination treatment), in-patient mortality remains unacceptably high (~10%), and unlikely to improve without wider implementation of pre-referral artemisinin ^49^ and better supportive treatments ^50, 51^. The overarching aim of the SMAART consortium was to find ways to improve outcomes from severe malaria by conducting better research studies faster. At an inception meeting in 2018 the current treatment guidelines were reviewed and found that most recommendations in the current WHO guidelines for the management of severe malaria are based on expert opinion, including controversial recommendations regarding several seemingly simple elements such as treatment thresholds for transfusion or correcting hypoglycaemia ^27^. The paucity of clinical trial data was stark given the substantial contributions of severe malaria to child mortality. The SMAART group considered both high priority risk factors (see Table 2) and missed opportunities to improve short and long-term outcomes which could be prioritised based on proof-of-concept Phase II trials. The group identified several high-priority candidates for testing in Phase I and II trials, targeting treatment and complications with high mortality, with primary endpoints based on mechanisms of action.

**Table 2.** Admission features and complications of malaria.

Admission feature or complication	Frequency	In-hospital Mortality* (Artesunate-arm) ^1^	
Coma	32–35%	18%	
Metabolic acidosis (base excess<-8 or lactate>5mmol/L)	43–44%	15%	
Renal impairment (Urea/BUN > 20 mmol/L)	24%	22%	
Hypoglycaemia (blood glucose <3 mmol/L)	10%	15% ^52^	
Convulsions	30–32%	14%	
Invasive bacterial co-infection	5.5%	24% (Meta-analysis) ^53^	
Blackwater Fever (region specific)	14–21%	Day-28 mortality 12% ^54^	
Recent or ongoing trials	Frequency	Mortality	Trial (reference)
Shock (mortality = no-bolus arm)	12%	8.5%	FEAST: 55
Severe anaemia	29–30%	1%	TRACT: 56, 57
Hypoxaemia (<90%)	15%-17%	2–13%	COAST: 58

Cerebral malaria was identified by the groups as a complication of severe malaria that has witnessed little change in outcome despite introduction of artesunate. Trials of osmotherapy, steroids and seizure prophylaxis as adjunctive therapies have failed to show any benefit and in some have resulted in worse outcomes in the interventional group.

Two key complications were considered as potential targets for future invention trials. The first was the most common terminal clinic event: respiratory depression and arrest. The second was repeated generalised tonic-clonic seizures which often precipitate the agonal event due to ‘spikes’ in intracranial pressure.

In the absence of mechanical ventilation there are very few options. High Flow nasal therapy and Continuous Positive Airway Pressure (CPAP) devices which apply positive pressure throughout the entire respiratory cycle in patients breathing spontaneously. However, for patients in whose respiratory efforts are compromised BCV applies both negative and positive pressure to the chest, covering both inspiration and expiration phases of breathing, which is more appropriate for periods of hypoventilation and apnoea. Moreover, the BCV is a NIV system that is portable, requires no intubation, does not require specialist medical personnel, and can be used across a wide weight and age range. BCV could be transferred to Africa if studies showed that it could be safely and effectively implemented.

The group consider the currently available ASMs for seizure prophylaxis and chose levetiracetam owing to its favourable pharmacological properties and good safety record – with minimal adverse events relating to respiratory depression. Seizure prophylaxis was considered for all children who have neurological presentations including those with a history of seizures since further seizures are harmful and risk the development of coma and difficulty in breathing. We therefore have modified the original proposal (see protocol versions section) to extend it to those with seizures and prostration (inability to sit upright without assistance in children over 8 months). Respiratory support with combined BCV and seizure prophylaxis was considered only relevant to those with coma.

In summary, to address a therapeutic gap in the management of cerebral malaria, we will explore the potential benefits of negative pressure ventilation (NPV) in combination with levetiracetam as a prophylactic ASM for preventing respiratory arrest and/or preventing or reducing intracranial hypertension by targeting the cerebral vasodilation, as a promising strategy to avert deaths and neurological impairments in children with cerebral malaria. We will also explore levetiracetam as a prophylactic ASM in children who are admitted with seizures in this illness.

## Trial status

The trial commenced enrolment on January 22, 2020, with the initial participant enrolled on February 3, 2020. However, due to the COVID-19 pandemic, study activities were suspended on March 20, 2020, following directives from the Ministry of Health aimed at curbing the spread of the disease. Subsequently, study activities resumed after a Site Initiation Visit (SIV) conducted on December 12, 2021, and officially recommenced on January 3, 2022. The trial is presently active and ongoing.

## Protocol version changes

The original Initial protocol submitted to SERU version 1.0 date 13 ^th^ April 2018 following the updates recommended by the reviewer, version 1.0 dated 19 ^th^ June 2018 was finally approved.

The regulatory PPB approved the international protocol version 1.0 dated 19 ^th^ June 2018.

In version 1.1 we requested a change in the monitoring device for CO2 to a non-invasive end-tidal expired CO2 monitoring rather than blood gases. This was approved on 25 ^th^ February 2019.

In Version 1.2 a change in project manager and data manager were requested. This was approved June 16 ^th^ 2020.

An amendment to change the primary endpoint from ECG recorded seizure to clinically reported seizure included in version 2.0. This was approved on 18 ^th^ July 2022 and subsequently approved by ICREC. The current approved version 2.0 in use.

## Roles and responsibilities

### Roles of the sponsor and funders

The sponsor and funder played no role in in study design and will play no role in data collection, trial management, analysis and interpretation of data and manuscript preparation the decision to submit the report for publication.

We thank United Hayek Industries, London who provided the BCV and consumable for the trial at reduced price.

### Trial management group

Kathryn Maitland, Emmanuel Oguda, Christabel Mogoka, Roisin Connon and Elizabeth C George

### Safety review committee

Prof. Timothy Peto (Chair): University of Oxford; Prof Jim Todd: London School of Hygiene and Tropical Medicine; and Dr Jane Crawley: University of Oxford.

### Sponsor

Imperial College London is the main research Sponsor for this study. For further information regarding the sponsorship conditions, please contact the Head of Regulatory Compliance at:

Joint Research Office, Room 221b, Medical School Building, St Mary’s Campus, Norfolk Place, London, W2 1PG. Telephone: +44 (0) 020 7594 1872.

## Ethics and consent

Ethical approval has been obtained from Kenya Scientific and Ethics Review Unit (SERU), Nairobi Kenya on 13 ^th^ July 2018 (protocol number RES/7/3/1), Imperial College Research Ethics Committee (ICREC) on 19 ^th^ June 2018 (protocol number 18IC4511). The trial was registered on the ISRCTN registry protocol number ISRCTN76942974 on the 5 ^th^ February 2019 and on the Pan African Clinical Trials PACTR number PACTR202112749708968 20th December 2021. Prospective written, informed consent will be sought from parents or guardians of children who are considered to be sufficiently stable.

## Data Availability

No data are associated with this article. Imperial College Research Data Repository: NOVICE extended data. https://doi.org/10.14469/hpc/14060 ^59^. Imperial College Research Data Repository: SPIRIT checklist for “Non-invasive Ventilation In Children with Cerebral Malaria Trial: A Phase I Safety and Dose finding trial (NOVICE-M Trial)”. https://doi.org/10.14469/hpc/13898 ^60^, Data are available under the terms of the Creative Commons Zero "No rights reserved" data waiver (CC0 1.0 Public domain dedication).
