# Supplementary material for: A Phase I trial of Non-invasive Ventilation and seizure prophylaxis with levetiracetam In Children with Cerebral Malaria Trial (NOVICE-M Trial)
Source: Wellcome Open Res. 2024 May 20;9:281. [Version 1] doi: 10.12688/wellcomeopenres.21403.1 (PMC11342035; doi:10.12688/wellcomeopenres.21403.1)
Supplement: Peer review history

# Reviewer report: Reviewer response for version 1

**DOI:** 10.21956/wellcomeopenres.23671.r91508

**Published:** 2024-08-22

**Authors:** Collins Ouma, Dr. Bernard Guyah

**Affiliations:** 1 Biomedical Sciences and Technology, Maseno University, Kisumu and Siaya, Kenya; 2 Maseno University, Maseno, Kisumu County, Kenya

Maitland et al. presents a protocol to conduct a Phase I trial in Kilifi, Kenya designed to provide data on safety, feasibility and preliminary data on seizure control using prophylactic ASM (levetiracetam) and BCV as non-invasive ventilatory support in children with cerebral malaria. Children aged 3 months to 12-years hospitalised with P falciparum malaria (positive rapid diagnostic test or a malaria slide), a Blantyre Coma Score ≤2 and a history of acute seizures in this illness are eligible for the trial. 

 I have the following comments for this particular protocol:

 1. What are the standard operating procedures for administering the interventions, and how will consistency be maintained throughout the trial?

 2. Would the sample size of 30 (distributed into N=10 per group for 3 groups) be adequate? Was there any sample size calculations for this trial?

 3. How will you ensure that staff are adequately trained in both the intervention and in monitoring participants to minimize bias?

 4. What logistical challenges might arise, and how will they be addressed?

 5. How will adherence to the intervention protocols be monitored?

 6. How will you define and measure tolerability for both non-invasive ventilatory support and LVT prophylaxis?

 7. What baseline data will be collected to compare against potential side effects during the trial? And how will these be monitored?

 8. How will you assess the impact of the interventions on the quality of life of participants?

 9. What known risks will be communicated to participants, and how will the study be designed to minimize these risks?

 10. How will safety data be reviewed and by whom? 11. What measures will be taken to protect vulnerable populations involved in the trial?

 12. What data are critical to collect in this Phase I to refine and optimize the design of subsequent trials?

 13. What is the contingency plan for unexpected challenges or deviations from the study protocol?

 14. What statistical methods will be used to analyze the safety and tolerability data?

 Thanks.

Is the study design appropriate for the research question?

Yes

Is the rationale for, and objectives of, the study clearly described?

Yes

Are sufficient details of the methods provided to allow replication by others?

Yes

Are the datasets clearly presented in a useable and accessible format?

Not applicable

Reviewer Expertise:

Public health, immunology, molecular biology, infectious diseases, NCDs

We confirm that we have read this submission and believe that we have an appropriate level of expertise to confirm that it is of an acceptable scientific standard.

# Reviewer report: Reviewer response for version 1

**DOI:** 10.21956/wellcomeopenres.23671.r91511

**Published:** 2024-08-13

**Authors:** Alexuse Saidi

**Affiliations:** 1 Kamuzu University of Health Sciences, Blantyre, Malawi

The study is clear however the following needs to be looked into:

- One inclusion criterion is that the client should either be RDT positive **OR** malaria slides positive. RDT can come out positive despite the parasites completely being cleared out of the human systems.
- What is the difference between "admission blood" and "the usual blood samples" under the sampling strategy?
- According to the design, children will be required to be contacted for review on days 28, 90, and 180, where are these days counted from? is it from the admission date (day 0) or the discharge date?
- On table one, under Laboratory tests, what will be the criteria for CM client to be discharged? I asking this question because Laboratory tests have been indicated to be sampled only at admission, after 8 hours, and after 24 hours. What will happen if there will still be some parasites after 24 hours?
- Under medical research ethics, if a client has withdrawn from participation, it is not appropriate to use previously collected samples or their corresponding samples.
- This study document was reviewed on 13th April 2018 as well as on 19th June 2018 so the document version number is supposed to change.

Is the study design appropriate for the research question?

Yes

Is the rationale for, and objectives of, the study clearly described?

Yes

Are sufficient details of the methods provided to allow replication by others?

Yes

Are the datasets clearly presented in a useable and accessible format?

Not applicable

Reviewer Expertise:

Malaria Pathogenesis

I confirm that I have read this submission and believe that I have an appropriate level of expertise to confirm that it is of an acceptable scientific standard.
